# Response to Difficulty Drives Variation in IQ Test Performance

**DOI:** 10.1162/opmi_a_00127

**Published:** 2024-03-26

**Authors:** Samuel J. Cheyette, Steven T. Piantadosi

**Affiliations:** Department of Brain & Cognitive Sciences, MIT; Department of Psychology, UC Berkeley

**Keywords:** IQ, Bayesian modeling, individual differences

## Abstract

In a large (*N* = 300), pre-registered experiment and data analysis model, we find that individual variation in overall performance on Raven’s Progressive Matrices is substantially driven by differential strategizing in the face of difficulty. Some participants choose to spend more time on hard problems while others choose to spend less and these differences explain about 42% of the variance in overall performance. In a data analysis jointly predicting participants’ reaction times and accuracy on each item, we find that the Raven’s task captures at most half of participants’ variation in time-controlled ability (48%) down to almost none (3%), depending on which notion of ability is assumed. Our results highlight the role that confounding factors such as motivation play in explaining individuals’ differential performance in IQ testing.

## INTRODUCTION

**Figure 1.**
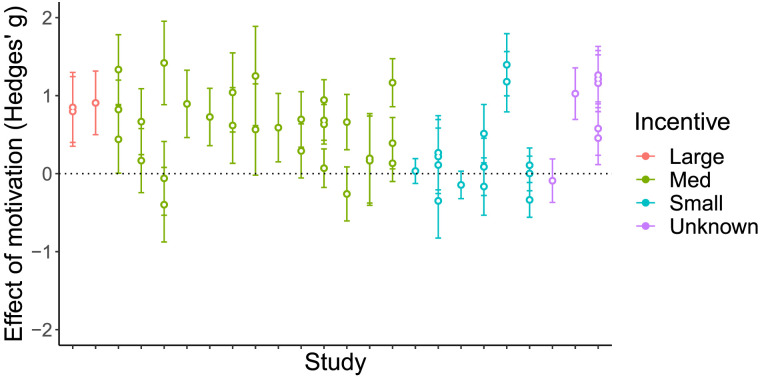
A visualisation of data from Duckworth et al. (2011) showing the effect size of a pay manipulation on IQ tasks (*y*-axis) across studies (*x*-axis), broken down by reward size (color). This robustly shows effect of pay manipulations on test outcomes.

Intelligence tests are central to many areas of applied and theoretical psychology, however the question of what IQ tests measure has been debated for decades (Ceci, 1996; Flynn, 1987; Gould, 1996; Jensen, 1998; Richardson, 2002; Mackintosh, 2011; Mensh & Mensh, 1991; Schönemann, 1983). Large and robust effects of coaching, schooling, practice, and pay (Briggs, 2001; Brinch & Galloway, 2012; Cahan & Cohen, 1989; Cliffordson & Gustafsson, 2008; Duckworth et al., 2011; Kulik, Bangert-Drowns, et al., 1984; Kulik, Kulik, et al., 1984; Powers, 1993; Ritchie & Tucker-Drob, 2018) on IQ test performance demonstrate that individual experiences and incentives affect test outcomes, independent of intellectual ability. Experiments that manipulate the amount of reward provided to participants based on performance find substantial, robust effects on test performance. Figure 1 shows data replotted from Duckworth et al. (2011)’s meta-analysis of prior pay manipulations, showing the overall effect of pay (left) as well as the pay broken down by coding of reward size (color). This illustrates a robust effect (Hedges *g* ≈ 1 or roughly 15 IQ points in the best cases) that appears sensitive to the amount of extrinsic reward.

**Figure 2.**
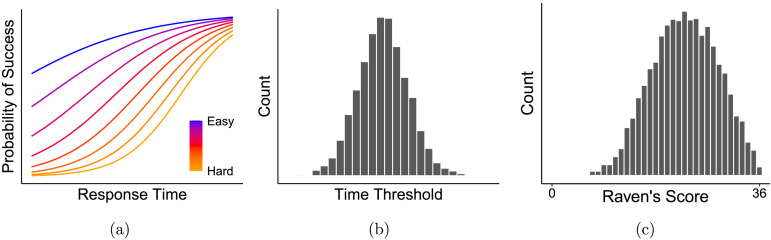
An illustration of the potential issue with uncontrolled variation in response times. (A) The conceptual tradeoff between an item’s difficulty (colors), the time taken on the task (*x*-axis), and the probability of responding accurately (*y*-axis). (B) Simulated participants with variation in their maximum time investment on a given question. (C) Simulated Raven’s scores given only the RT-accuracy curves and natural variation in participants’ response time thresholds shown in the other two panels.

While these results show that individuals will change their performance in response to external incentives, they do not demonstrate that people vary *intrinsically* in the effort and strategies they bring into testing situations. This possibility is important for understanding the construct validity of IQ tasks because individual variation in intrinsic effort or strategy would masquerade as differences in ability. Specifically, the speed-accuracy tradeoff that each individual decides upon should be expected to impact their performance. This possibility was highlighted by early experimental psychologists like Thurstone (1937), who articulated the inevitable tradeoff between accuracy and time in testing situations. Figure 2A shows a sketch of the relationship between accuracy (“probability of success”), time, and difficulty highlighted in Thurstone (1937), capturing the idea that difficult items will tend to take more time to achieve a high probability of success. The interrelationship highlights that a finding that time investment differs between individuals is relevant to measuring ability: a person’s ability—perhaps quantified as difficulty at a fixed level of accuracy and RT—cannot be read off of their performance if individuals differ in time investment. Figure 2B and C illustrate this point: assuming a fixed level of ability across a population, natural variation in the maximum time participants’ allot to a question (2b) could lead to substantial variation in Raven’s scores (2c).

For this reason, it is unclear to what extent the positive manifold reported in intelligence research since Spearman (1904) might be explained not through a shared component of intellectual capacity, but through a shared component of effort or time investment in testing tasks. This idea has received surprisingly little attention in psychology’s IQ debates (Goldhammer & Entink, 2011; Goldhammer et al., 2015; Scherer et al., 2015; Tate, 1948; Thissen, 1976). A notable exception is the work of Thissen: following Furneaux (1973), Thissen (1983) showed a correlation of *R* = 0.94 between slowness and outcome on Raven’s tasks (Raven, 2000; Raven et al., 1989). Thissen concluded that “working slowly and carefully is strongly related to the probability of responding correctly, and what is measured is largely slowness.”

It is important to distinguish the idea that performance depends on slow, careful, sustained attention and effort from another popular hypothesis in psychometrics. A considerable body of work has examined how *general* processing speed fits into the picture of psychometric *g* (Bates & Stough, 1997, 1998; Carroll, 1993; Evans & Deary, 1994; Deary & Stough, 1996; Grudnik & Kranzler, 2001; Jensen, 1982, 1985, 2006; Kyllonen & Zu, 2016; Nettelbeck, 1998; Neubauer, 1990; Sheppard & Vernon, 2008; Vernon, 1983). Such work typically quantifies each individual’s processing speed on simple perceptual tasks like responding quickly to a light stimulus as in Hick (1952). This hypothesis is distinct from the idea explored by Thissen (1983) because the time spent on each question is dependent on higher-level cognitive processes than those involved in perceptual tasks. A considerable literature in test theory (Gulliksen, 1950; van der Linden, 2009) has examined the relationship between time-limits and performance broadly in testing situations (e.g., Bridgeman et al., 2004; Davidson & Carroll, 1945; Kyllonen & Zu, 2016; Rindler, 1979). This has resulted in proposed measures in psychometrics that combine speed and accuracy (Liesefeld et al., 2015; Liesefeld & Janczyk, 2019; Townsend & Ashby, 1983; Vandierendonck, 2018), or jointly analyze both (Bolsinova et al., 2017; De Boeck & Jeon, 2019; Entink et al., 2009; Kyllonen & Zu, 2016; van der Linden & Fox, 2016; van der Linden et al., 2010). Such tradeoffs are even attested in other species (Bogacz et al., 2010; Chittka et al., 2009; Goldhammer, 2015; Heitz, 2014; Heitz & Schall, 2012; Luce, 1986; Wickelgren, 1977). Yet, in the context of IQ testing, it is standard to compute overall accuracy, and not even look at timing patterns, much less control them.

Here, we build on Thissen (1983) to examine the relationship between individuals’ response times across questions (reflecting strategy and effort) and overall test performance in a Raven’s task. We aim to update these results with modern methods, including Bayesian data analyses that control for items and participants, large sample sizes, and pre-registered experimental designs and data analysis, and then interpret these findings in the context of the construct validity for these tasks. Several behavioral patterns are possible as items become more difficult throughout a Raven’s task: (i) participants could spend more time on *more* difficult items, likely exerting greater effort in order to achieve high accuracy; (ii) participants could spend *less* time on difficult items, perhaps meta-cognitively realizing that a given problem is out of reach; or (iii) participants could be relatively insensitive to item difficulty, perhaps allocating time or effort equally throughout the test. Crucially, participants may show different patterns of behavior across questions, and our analysis aims to determine how variability in these patterns affects their overall score.

## EXPERIMENT

### Method

We pre-registered an experiment where 300 participants took an online version of Raven’s Progressive Matrices (Raven, 2000) in September of 2022.^1^ The experiment was run on Prolific, which has been found to yield higher-quality data than other online platforms (Peer et al., 2022). As is standard for this task, participants were told to complete as many of the items as they could in the maximum time of 40 minutes. Participants received compensation of $7.50 for completion of the task. They were given instructions adapted from the 1988 Advanced Progressive Matrices manual (Raven & Court, 1988) for use in an online study. Unlike standard analyses of this task which focus on overall accuracy, we recorded response time for each item. These response times reflect either how long it took participants to find a solution, or how long they were willing to spend on a given item before moving on. Following our pre-registration plan, which was determined through a smaller pilot experiment on an independent pool of participants, we removed participants whose median response time was less than 10 seconds. This left 276 total participants in our main analysis. We *z*-scored RT across all participants in order to use a standardized scale, but maintain intrinsic variation between individuals. We also collected data on participants’ demographics and socioeconomic status (e.g., income and education), and asked participants to report how many questions they thought they correctly answered.

### Results

**Figure 3.**
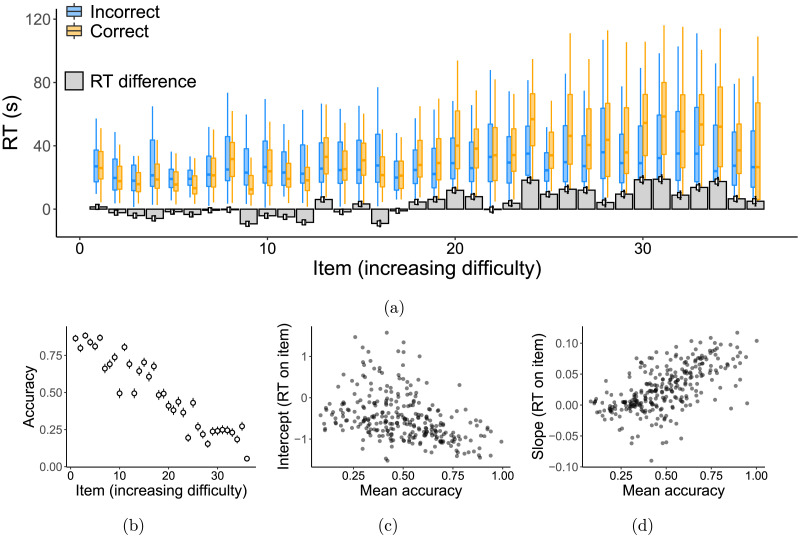
(A) Boxplots showing the distribution of response times to each question, grouped by accuracy (color). The gray bars depict the mean response time difference between correct and incorrect respondents. (B) Validation of the task, showing generally decreasing accuracy for harder items. (C–D) Inferred intercepts (C) and slopes (D) in a regression item (e.g., difficulty) and RT, separately for each subject, plotted against participants’ mean accuracy across Raven’s problems.

Aggregate response times are show in Figure 3A, which show the RT for each item throughout the task, grouped by accuracy. Participants tended to spend more time on difficult (later) questions, but this effect is primarily driven by those who answer correctly: participants who are incorrect on later questions don’t tend to spend more time on them. Differential time investment on hard questions hints that individuals may tend to be inaccurate when they choose to invest less time in a problem. One way to see whether subjects participant—and whether any variation is associated with accuracy—is to run a regression *within* each participant predicting their RT from the item number, using item number as a proxy for difficulty. Figure 3C and D shows these coefficients for each subject (*y*-axis) plotted against their overall task performance (*x*-axis). Participants who performed well tended to spend more time working on the later (harder) questions.

**Figure 4.**
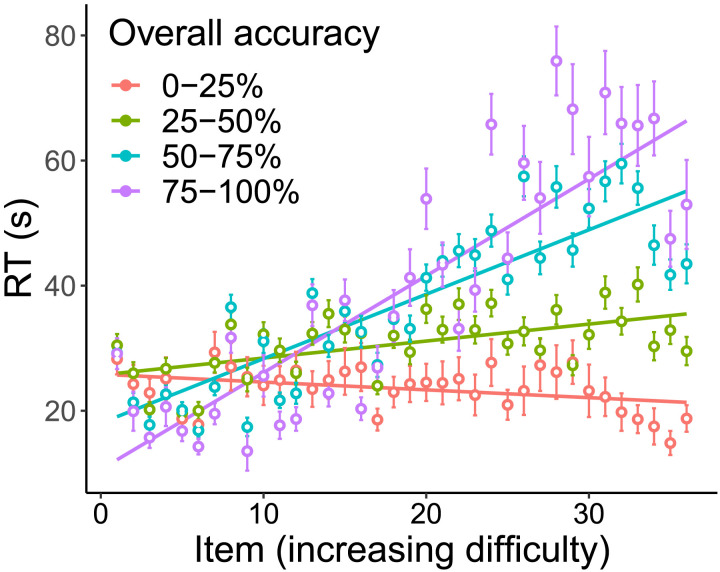
Response time (*y*-axis) as a function of problem number (*x*-axis), which is treated as a proxy for difficulty. Participants are grouped (color) according to their overall accuracy on the test. Participants who performed well were the ones who invested the most time on the harder problems.

An aggregate view of this effect is shown in Figure 4, which plots the relationship between RT and accuracy for participants, broken down by their overall accuracy. This figure paints a clear picture that those who performed well on the task tended to spend more time on the harder questions. The effect size between groups is huge: the best-performing quartile of participants spend approximately *three times* as long on the hard questions as the lowest-performing quartile. We emphasize that everyone was given the same instructions on the same task, so these differences represent *intrinsic* variation in how individuals approach the task. Individual subject plots can be found in the SI, and demonstrate a similar pattern.

Both intercepts and slopes are statistically correlated with overall Raven’s (*R* = –0.34, *p* < 0.001 and *R* = 0.65, *p* < 0.001, respectively). Partialing the variance in overall performance between slope and intercept coefficients, we find Raven’s score much more likely reflects response to difficulty (slope partial *R*^2^ = 0.36) as opposed to average amount of overall time spent (intercept partial *R*^2^ = 0.01). This indicates that these differences in slope matter to overall performance and thus the difficulty-time slope confounds Raven’s measures which use overall performance. Following our preregistration plan, we also quantified variation in subject responses to difficulty by comparing two regression models that predicted RT: one where slopes by item were allowed to vary by subject and one where they were not. Both regressions included coefficients for item and accuracy. This revealed strong evidence in favor of the model that varied slopes by participant (ELPD difference 627.9 with a standard error of 38.5), providing further statistical support to the idea that individuals in a Raven’s task respond differently to difficultly.

These differing slopes raise the natural question of how individuals might have performed *if* they had allocated time differently. Such a counterfactual is a step towards quantifying “ability” because it targets a subject’s potential—what they could do—rather than what they happened to decide to do in our task. However, it is only a partial step towards ability because it leaves other factors like motivation and coaching uncontrolled. Following our pre-registration plan, we constructed a joint, fully Bayesian data analysis of accuracy, RT, and the latent difficulty of each item. One way this model differs from the previous regression is that the latent difficulty of each item is assumed to affect response time, rather than item number as above (which is only imperfectly correlated with difficulty). By including RT, this model goes beyond recent item response theory models of the same task (Bürkner, 2020; Myszkowski & Storme, 2018); it differs from Thissen (1983) in that it uses a Bayesian analysis (Fox, 2010) that is hierarchical, allowing us to extract confidence in each individual subject and item parameter, while optimally reducing estimation noise through the use of partial pooling (Gelman & Hill, 2006). The model predicted the z-scored RT of the *s*’th subject on the *i*’th item, *R*_*si*_ as

$$R_{si}∼\text{Normal}\left(\beta_{0}+\beta_{0s}+\left(\beta_{1}+\beta_{1s}\right)\cdot d_{i},\sigma \right)$$ (1)

where *d*_*i*_ is the latent difficulty of the *i*’th item. Here, *β*_0_ and *β*_1_ are the overall subject intercept and slope, which are given *Normal*(0, 3) priors. *β*_0*s*_ and *β*_1*s*_ are the *s*’th subject’s adjustments to those means, which are assumed to follow *Normal*(0, *ν*) for *ν* ∼ *Exponential*(1). The item difficulties were given a prior *d*_*i*_ ∼ *Normal*(0, 1). The standard deviation of response times was given prior *σ* ∼ *Exponential*(0.1).

Simultaneously with (1), the probability of responding correctly for subject *s* on item *i* (*P*_*si*_) was modeled in a hierarchical logistic setup, such that

$$\text{logit}\left(P_{si}\right)=\left(\left(\gamma_{0}+\gamma_{s0}\right)+\left(\gamma_{1}+\gamma_{s1}\right)\cdot R_{si}+\left(\gamma_{2}+\gamma_{s2}\right)\cdot d_{i}+\lambda_{1}\cdot \beta_{0s}+\lambda_{2}\cdot \beta_{1s}+\lambda_{3}\cdot \beta_{0s}\cdot \beta_{1s}\right),$$ (2)

where:

*γ*_0_ + *γ*_*s*0_ = subject accuracy intercept
*γ*_1_ + *γ*_*s*1_ = effect of response time (*R*_*si*_) on accuracy
*γ*_2_ + *γ*_*s*2_ = effect of item difficulty (*d*_*i*_) on accuracy
*λ*_1_ = effect of overall time investment (*β*_0*s*_) on accuracy
*λ*_2_ = effect of RT response to difficulty (*β*_1*s*_) on accuracy
*λ*_3_ = interaction between overall time investment (*β*_0*s*_) and response to difficulty (*β*_1*s*_) on accuracy

Response accuracy was then distributed according to *A*_*si*_ ∼ *Bernoulli*$\left(P_{si}+\frac{1}{8}\cdot \left(1-P_{si}\right)\right)$, where the $\frac{1}{8}$ · (1−*P*_*si*_) term represents the probability of correctly answering a question by guessing randomly.

Here, *γ*_0_, *γ*_1_, *γ*_2_, *λ*_1_, *λ*_2_, *λ*_3_ are group mean parameters and were given *Normal*(0, 3) priors. The *γ*_*s*·_ are individual subject parameters that, as with the *β*_*s*·_ parameters, were drawn from *Normal*(0, *ν*) for *ν* ∼ *Exponential*(1). When combined with (1), this form of model can be thought of as inferring latent participant measurements, *β*_0*s*_ and *β*_1*s*_, that characterize how each person responds to difficulty, which are then used as predictors of accuracy, in addition to the RT on each item *R*_*si*_. The net effect is that the other accuracy predictors (e.g., *γ*_0*s*_, *γ*_1*s*_, *γ*_2*s*_) are then controlled for the patterns of response to difficulty apparent in RT. This hierarchical setup allows each subject estimate to be informed by the others, but also permits individual variation through the subject-specific parameters. The model was run using Stan (Carpenter et al., 2017), with 4 chains and 5,000 samples of its NUTS sampler (Hoffman & Gelman, 2014). Convergence was assessed using traceplots and $\hat{R}$ values, which were less than 1.01.

**Figure 5.**
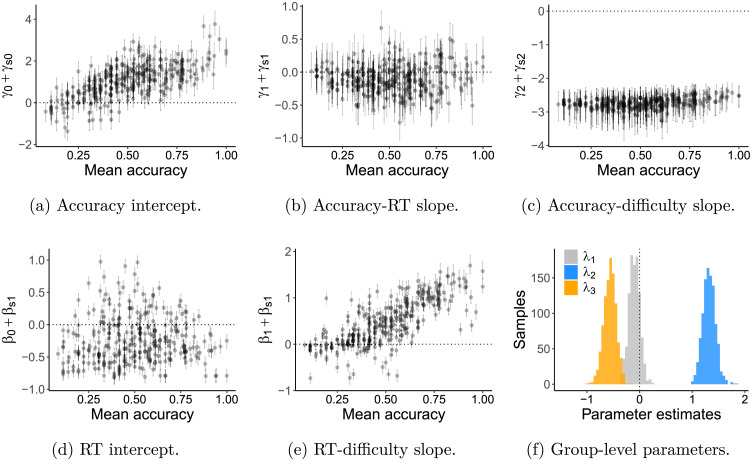
Model parameters predicting RT and accuracy. (A–E) Each point represents a single subject, and their parameter value (*y*-axis) is plotted against their overall Raven’s accuracy (*x*-axis). (F) Group-level posterior estimates for *λ*_1_, *λ*_2_, and *λ*_3_.

Figure 5 shows the inferred individual parameters from this model as a function of each subject’s raw accuracy on the Raven’s task (*x*-axis). This provides several intuitive checks that the model is working appropriately—for example, Figure 5C shows that participants tend to be less accurate on more difficult problems since these values are negative. Figure 5E, giving the RT response to difficulty, replicates the analyses above to show that high-performing participants also tended to spend more time on more difficult problems. There are also many participants who had negative or essentially zero difficulty slopes for RT, meaning that they do not spend more time on harder problems. These people tended to perform least well overall. However, the RT intercept (time at average difficulty) in Figure 5D was relatively unrelated to overall correctness, showing that the effects are mostly about response to difficulty rather than starting time investment. Interestingly, the participants with the highest performance overall did *not* show better accuracy slopes (Figure 5B), meaning that their accuracy-per-time-invested did not improve faster than others. However, their accuracy intercepts (Figure 5A) did tend to be higher, which is almost inevitable in this kind of model. Figure 5F shows the values of *λ*_1_, *λ*_2_, *λ*_3_ showing that participants who had higher *β*_*s*1_ tended to be more accurate.

It is important to note that differences in participants’ accuracy intercepts under this model may, and indeed likely do, reflect many other factors than just ability. That is, the intercepts simply reflect all the remaining variance from the model not explained by reaction time, since we were not measuring or controlling other differences in the model. Familiarity with similar tests, for instance, could explain part of the variance in accuracy that is reflected as differences in participants’ intercepts. In fact, participants who responded that they had taken a similar test scored 3.3 points (20%) higher, on average, than participants who reported that they had not taken a similar test (*F*(1, 269) = 16.27; *p* < 0.001).^2^ Since there was no relationship between RT and having taken a previous test (*F*(1, 269) = 0.1; *p* = 0.74), this portion of variance (about 6%) is simply incorporated into participants’ intercepts. This is of course true as well for the myriad other factors that are not correlated or imperfectly correlated with RT, such as focus.

With that note of caution in mind, the model can still be used to estimate measures of performance controlled both for response time and pattern of response to difficulty, which can provide an upper bound on how well Raven’s can quantify ability. The posterior median accuracy intercept quantifies the accuracy that participants would have at the mean RT, with the mean response to difficulty, on the easiest items. This is correlated *R* = 0.67 with overall Raven’s score. The posterior median average difficulty at which people would be 50% accurate at the average RT is correlated *R* = 0.69 with overall Raven’s score. Third, the posterior median time, according to the model, it would take someone to solve the most difficult problem is correlated at *R* = 0.17 with overall Raven’s score. This means that, depending on which upper-bound of “ability” we think is the most appropriate formalization, Raven’s tasks capture at most approximately *half* (*R*^2^ = 0.695^2^ = 0.48) of the subject variation in time-controlled ability, and possibly down to virtually none (*R*^2^ = 0.174^2^ = 0.03).

### Re-analysis with Higher-performing Participants

One potential objection to our findings is that, because the experiment was conducted online and without great incentive to perform well, a significant subset of participants may not have been engaged—more than would be expected in traditional test-taking settings—and these participants are driving all the results. It is true that participants in our sample performed somewhat worse on average on our task than in samples reported in the APM manual (Raven & Court, 1988): 52% correct in our sample vs. 53–58% correct in the APM depending on the population tested. To account for the possibility that lower average engagement levels were distorting our results, we ran post-hoc (not pre-registered) analyses using an even stricter exclusion criteria. Specifically, anyone who did not answer all of the first three questions and at least 25% of all questions correctly was excluded. This left 176 participants who answered 58% of the questions correctly on average.

Even in this higher-performing sample, we find that differential time investment on difficult questions is a strong predictor of overall performance. We first re-ran the regressions within individual participants, predicting their response time from item number. Both intercepts and slopes were again correlated with overall Raven’s (*R* = −0.49, *p* < 0.001 for intercepts, *R* = 0.66, *p* < 0.001 for slopes); partialing the variance shows that Raven’s score is better explained by response to difficulty (slope partial *R*^2^ = 0.26) than by overall time spent (*R*^2^ = 0.01). We then re-ran the hierarchical Bayesian model and found, similar to the initial results, that the posterior median accuracy intercept explained about half of differences in overall score (*R*^2^ = 0.53) and the posterior median time required to solve the most difficult problem explained almost none (*R*^2^ = 0.01).

## DISCUSSION AND CONCLUSION

These results document substantial variation in how participants respond to difficulty in a standard intelligence task. Moreover, the variation matters: participants’ response to difficulty explains 42% of the variance in overall performance. In this case, it is not surprising that a measure like Raven’s would correlate with other life outcomes (Mackintosh, 2011; Richardson et al., 2012; Strenze, 2007), just as personality measures do (Duckworth & Seligman, 2005; Duckworth et al., 2019; Heckman & Kautz, 2012; Poropat, 2009). The idea that time investment on Raven’s might drive correlations with life outcomes is conceptually close to “grit” (Duckworth et al., 2007; Duckworth & Quinn, 2009), which is an individual measure intended to capture an individual’s willingness to work towards a long-term goal (for critiques, see Credé et al. (2017)). Notably, it was not the faster (or slower) workers or thinkers who did well, but rather those who dedicated more time to the hard questions.

An important question is how much the results from our study—which used an online ‘crowd labor’ marketplace to recruit participants—will generalize to a traditional test setting. In particular, online platforms may incentivize strategic time allocation, and therefore have a greater time-difficulty tradeoff, relative to an in-person test. However, we believe that our findings have broad applicability, and are likely to generalize, for several reasons. First, recent studies have found that Prolific participants generally exhibit high levels of task engagement, supported by strong scores on tests of attention, comprehension, and reliability (Peer et al., 2022). Second, our re-analysis of high-scoring participants yielded results that were remarkably consistent with the entire sample, suggesting that even within more motivated groups there are still large individual differences in responses to difficulty. Lastly, the growing preference for online platforms in social science research—due to their cost-effectiveness, demographic diversity, and generally high quality of data—underscores that, if nothing else, our findings are relevant to contemporary social scientists interested in individual differences.

Our results align with a recent study by Schirner et al. (2023), which found that participants in the Human Connectome Project who had higher Penn Matrix Reasoning scores were those who took longer on hard questions. They linked the differential time allocation to easy and hard problems to measures of functional connectivity, finding that slower solvers had higher resting state connectivity. Simulations from a network model, which represents relationships between brain regions and mutual patterns of excitation and inhibition, identified ratios of excitation and inhibition between regions as a plausible neural candidate underlying differences in functional connectivity and, they argue, the difference between high-*g* and low-*g* individuals. However, that work leaves explanations at a cognitive level largely unaddressed.

There are several possible drivers of the relationship between response to difficulty and success on reasoning tasks. First, people’s decisions about how much time to invest in each problem may be driven by meta-cognitive awareness or belief about their likelihood of finding the correct solution in a reasonable amount of time. Participants may give-up on questions they judge to be too difficult, and this may even reflect a sensible test-taking strategy, since the test has an overall time limit. However, very few participants (4%) ran out of time at the end, making it less likely that participants who invested less time on hard questions were using a rational strategy to maximize performance. Furthermore, while confidence is a well-known factor affecting test-taking (Ergene, 2003; Stankov & Lee, 2008; Stankov et al., 2012), differences in test strategy due to confidence is only weakly supported by our data: subjects’ overall score was correlated with a confidence rating they provided at the end (*R* = 0.52, *p* < 0.001), but their confidence was only weakly correlated (*R* = 0.17, *p* = 0.003) with the average *time* they spent on the task (i.e., explaining less than 3% of the variance in total test time). We note though that feedback was not provided in the task, so any person’s judgements about their own ability must come from intrinsic beliefs or suppositions about what the correct answers were or how easy they were to find.

A second, non-exclusive, possibility is that participants vary intrinsically in how much effort they are willing to invest in the task. When the reward size is not directly or obviously coupled to outcomes, participants may defaultly choose to invest variable amounts of time and energy. This idea is supported by the moderate to large effect sizes reviewed above for how task incentives affect performance (Duckworth et al., 2011). Such a finding has the potential to explain other demographic influences on Raven’s performance—for example, people with less schooling may be less familiar with or comfortable with testing situations and the sustained energy and attention they require; and people from lower-socioeconomic levels may intrinsically make a different tradeoff with their time.

Either possibility—rational meta-cognitive strategies or intrinsic variation in effort—is markedly different from the standard interpretation of IQ tests as providing a measure of “ability.” The notion that “intelligence” is what such tests quantify *by definition* has found some popularity (Boring, 1961; Van der Maas et al., 2014), but the view becomes difficult to sustain once alternative predictors of performance are clearly articulated. The amount of time someone allocates to a task is, we believe, not what anyone actually means by “intelligence.” Indeed, given variation in time investment, attempts to develop factor-analytic theories of intelligence seem doomed to uninterpretability: once the underlying measures are highly confounded by individual variation in effort or interest, the resulting factor structure means little. A way out of this is to focus on uncovering mechanisms and testing them empirically.

We emphasize that the amount of time spent on each item is likely only a proxy for real *cognitive* approaches to solving Raven’s tasks, and should not be confused for the real cognitive mechanism generating success in the task. For example, some authors have developed computational models which formalize mechanistic hypotheses about how intelligent agents may solve Raven’s or Raven’s-like problems (Depeweg et al., 2018; Gonthier & Roulin, 2020; Hernández-Orallo et al., 2016; Kunda et al., 2013; Little et al., 2012; Lovett et al., 2010; Carpenter et al., 1990), often searching over logical, rule-like representations, a recently popular approach to explaining high-level cognition (Rule et al., 2020). Other work has documented the effects of speed in generating possible rules (Verguts et al., 1999). Verguts and De Boeck (2002) showed that people’s search for rules preferentially re-uses rules they previously encountered—a finding which might provide a cognitive basis for practice and coaching effects. Carpenter et al. (1990) used eye-tracking and verbal reports from subjects engaged in a standard Raven’s task and showed that participants incrementally find components of rules, emphasizing the search process required to solve these problems. Work has shown that eye movements reflect different strategies for finding solutions (Hayes et al., 2011; Vigneau et al., 2006), and in fact that eye-movement differences may confound claimed processing time correlations (Bors et al., 1993).

A focus on understanding the real mechanisms of performance—developing models which can themselves solve problems like Raven’s—is a promising way to resolve the field’s century-long debate about the construct validity of intelligence measures. Timing decisions are one of the most basic components of mechanisms, but success is only possible when strategic decisions are combined with the right representations and inference procedures, which remain unclear. It is notable that neglect of mechanism has prevented the field from centering perhaps *the most basic* fact about such a widely used psychometric test: that the people who score highly are those who invest the most time on hard questions.

## FUNDING INFORMATION

This work was supported by a seed grant from the Institute for Brain & Cognitive Sciences at UC Berkeley to Samuel Cheyette & Steven Piantadosi.

## AUTHOR CONTRIBUTIONS

SJC & STP contributed equally to conceptualization, design, and writing. SJC led the data analysis with support from STP.

## DATA AVAILABILITY STATEMENT

Data and model code are freely available at https://osf.io/9rz2v/.

## Notes

^1^ The pre-registration, along with data and analysis, can be found at https://osf.io/9rz2v/.^2^ 7 participants declined to answer this question.

## Supplementary Material


