# Supplementary material for: Response to Difficulty Drives Variation in IQ Test Performance
Source: Open Mind (Camb). 2024 Mar 26;8:265–77. doi: 10.1162/opmi_a_00127 (PMC10990577; doi:10.1162/opmi_a_00127)
Supplement: Supplementary file 1 [file opmi-08-265-s001.pdf]

Supplementary Results

Demographics

Of the non-excluded participants, 120 were female, 150 were male, 5 were non-binary, and one declined to answer. The minimum age was 18, the maximum age was 91, and the median age was 34. We only included a coarse measure of income (9 bins from 0 to \$100,000 or more), but the median income was \$25,000 and the modal income was \$35,000, with 11 participants reporting making more than \$100,000 in the last year. There were 205 who identified as White, 27 Black, 18 Asian-American, 14 Mixed, 2 Native American, 1 Pacific Islander, and 6 Other (3 participants declined to respond). 36 participants had not gone to college or vocational school, 206 had gone to at least some college, and 32 had graduate degrees (2 declined to answer).

None of these demographic characteristics were strongly correlated with overall accuracy. There was not a significant difference between genders ($F(2, 272) = 2.49$; $p = 0.08$). There was a slight negative correlation between age and overall score ($b = -0.08$; $t = -2.65$; $p = 0.008$), with an overall R^2 of 0.025. There was no significant correlation between income and performance ($F(1, 257) = 0.33$; $p = 0.56$). There was a significant effect of race ($F(6, 266) = 2.35$; $p = 0.03$; $R^2 = 0.05$), but across t-tests with Bonferroni corrections there were no significant differences from any racial group with those identifying as White ($ps > 0.02$). To determine an effect of education, we collapsed the responses into four categories: high school or less, some college or vocational school, college, and advanced degree. Interestingly, there was not a significant effect of education on performance ($F(3, 270) = 1.75$; $p = 0.16$).

Posterior correlations of RT-intercept and RT-slope with accuracy

At the suggestion of a reviewer, we implemented a Bayesian version of the within-subject regressions in Stan, inferring response times from item number, which we then correlated across posterior samples

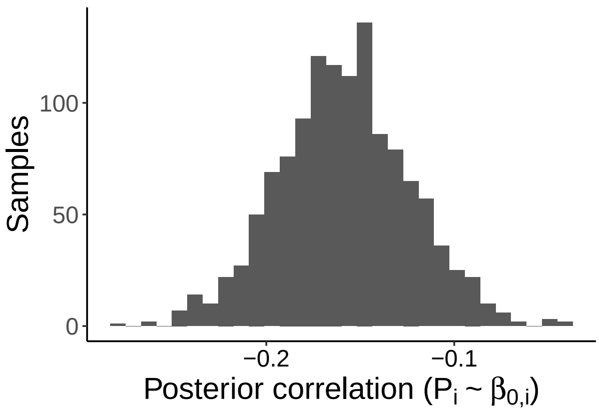

(a) Accuracy intercept.

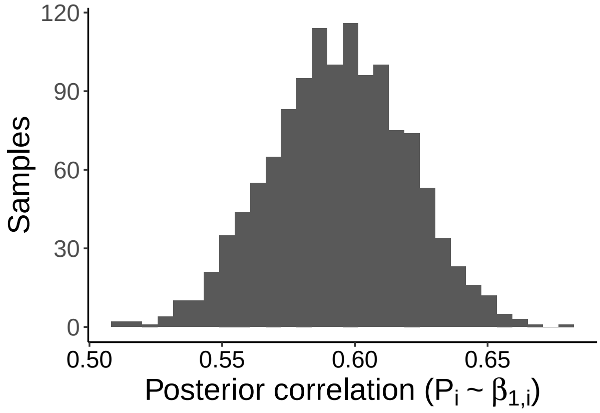

(b) Accuracy-RT slope.

Supplementary Figure 1: Histograms of posterior correlations between (a) RT-intercepts ($\beta_{0,i}$) and inferred mean accuracy from the model and (b) RT-slopes ($\beta_{1,i}$) and mean accuracy (P_i).

with overall accuracy (shown in the main text Figure 3c-d). Here, we use a hierachical setup to jointly infer group-level and subject-level RTs and a mean accuracies, which allows us to obtain credible intervals for the correlation between participants' RT intercepts and slopes with their overall performance. The model posits that each subject's RT (standardized across all subjects) and binary correctness of responses are influenced by subject-specific parameters and item characteristics. For each subject, a linear regression for RT is defined with coefficients $\beta_{0,i}$ and $\beta_{1,i}$, and a scale parameter $\sigma_{R,i}$ for the response variability. The correctness of responses is modeled as a Bernoulli process with a subject-specific mean accuracy parameter α.

The subject-specific parameters, $\beta_{0,i}$, $\beta_{1,i}$, $\sigma_{R,i}$, and P_i are drawn from group-level distributions. The group-level means of these parameters are denoted as μ_{β_0}, μ_{β_1}, μ_{σ_R}, and μ_P, while their group-level standard deviations are σ_{β_0}, σ_{β_1}, σ_{σ_R}, and σ_P. We set the priors for the group-level means as follows: $\mu_{\beta_0} \sim \mathcal{N}(0, 3)$, $\mu_{\beta_1} \sim \mathcal{N}(0, 1)$, and $\mu_{\sigma_R} \sim$ Exponential(0.1) for the response variability, while $\mu_\alpha \sim$ Beta$(1, 1)$ for the mean accuracy. The standard deviations σ_{β_0}, σ_{β_1}, and σ_{σ_R} are drawn from Exponential(1), and σ_P, acting as a concentration parameter for the Beta distribution of μ_P is drawn from Exponential(0.1).

We found that the median correlation from the posterior samples of participants' RT intercepts ($\beta_{0,i}$) with their mean accuracy α was -0.16, with a 95% credible interval of -0.23 to -0.09. The median correlation of participants' RT slopes with their mean accuracy was 0.60, with a 95% credible interval of 0.54 to 0.65. These numbers are similar to the point estimate r-values reported in the main text, though surprisingly slightly attenuated. The distribution over correlations is shown in Supplementary Figure 1.

Bayesian model without interaction parameter λ_3

Because the Bayesian model parameter λ_3, which represents the interaction between overall time investment (β_{0s}) and response to difficulty on accuracy is somewhat difficult to interpret, we re-ran

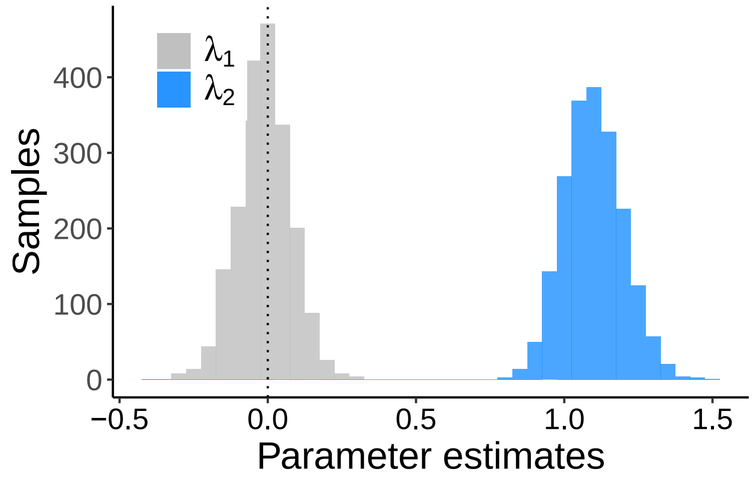

Supplementary Figure 2: Histograms of posterior samples of λ_1 and λ_2 from the Bayesian model without the interaction parameter λ_3.

the model without it. We would expect to see that λ_1, which represents the effect of overall time investment on accuracy would be close to zero still or slightly negative; and λ_2, which represents the effect of response to difficulty on accuracy, would still be positive. As Figure 2 shows, this is indeed the case. The median λ_1 is 0.0 (CI=[-0.19,0.16]) and the median λ_2 is 1.09 (CI=[0.92, 1.30]).

Inferred difficulty

Although Raven's problems are designed to get increasingly difficult throughout the test, we wanted to verify that the Bayesian model inferred this increasing difficulty as well. We examined the relationship between the posterior estimates of difficulty parameter d_i as a function of item number and found a median correlation of $r = 0.92$. This is shown in Supplementary Figure 3.

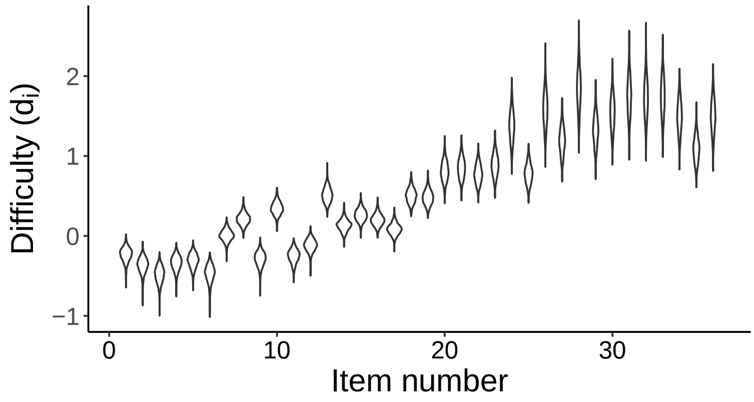

Supplementary Figure 3: Posterior of inferred item difficulty d_i as a function of item number.

Individual differences

Supplementary Figure 4 shows response times for each participant (panels) across each item in the test, grouped by whether they answered the question correctly (black/white color). The panels are arranged from lowest to highest RT-by-item slope, so that participants further to the top left have more negative RT slopes and participants further to the bottom right have more positive RT slopes.

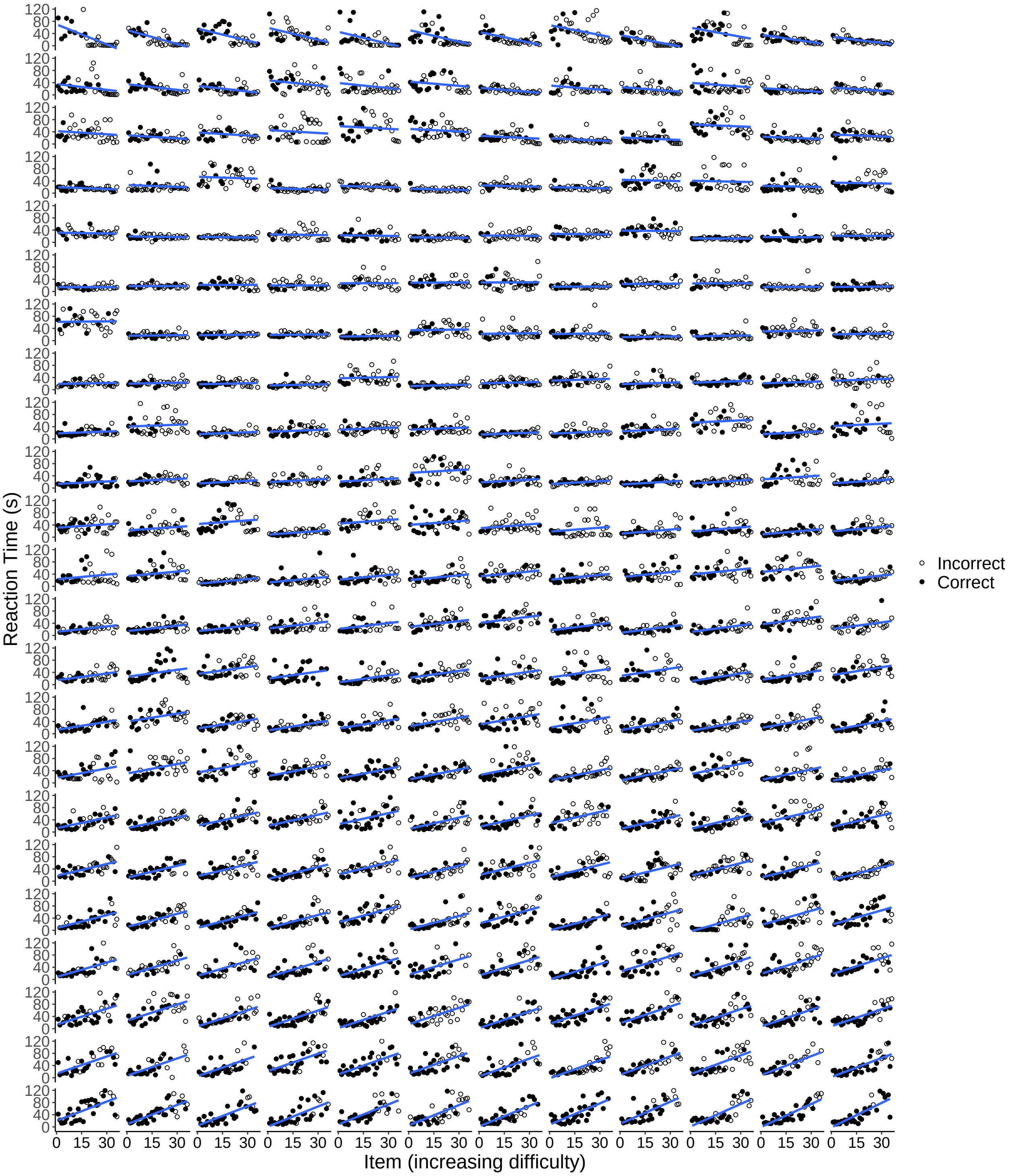

Supplementary Figure 4: The response times for each participant in the experiment across all items. The panels are arranged from lowest to highest RT by item slope.
